# Implementation of Modified Enhanced Recovery After Surgery Protocols for Elective Incisional Hernia Repair After Damage Control Surgery: A Retrospective Observational Study

**DOI:** 10.3389/jaws.2025.15474

**Published:** 2026-01-06

**Authors:** Marharyta Smirnova, Oleh Herasymenko, Mykhailo Koshikov, Vitalii Melnyk, Alim Ulukhanov

**Affiliations:** Military Medical Clinical Centre of the Southern Region, Odesa, Ukraine

**Keywords:** incisional hernia, ERAS, damage control surgery, enhanced recovery, opioid-freeanalgesia

## Abstract

**Background:**

The ongoing full-scale war in Ukraine has led to a significant increase in the number of patients undergoing damage control surgery following abdominal shrapnel wounds. These injuries are consistently associated with extensive soft tissue defects of the abdominal wall and secondary wound healing that frequently lead to the formation of large ventral hernias. In such patients, the primary goal is to provide the safest possible treatment and facilitate rapid recovery. The implementation of Enhanced Recovery After Surgery (ERAS) protocols has shown proven benefits in elective surgical settings. However, their use in ventral hernia repair remains insufficiently studied. The aim of this study is to evaluate the safety and effectiveness of adapted ERAS protocols in the management of ventral hernias after damage control surgery.

**Methods:**

This retrospective cohort study included 62 males divided into two groups based on the treatment period. Patients treated in the period before September 2024 received standard care (non-ERAS group), and those treated between September 2024 and April 2025 received treatment with implementation of ERAS protocols (ERAS group). All surgical procedures were performed using an open approach. Intraoperative and postoperative parameters were compared, including operative time, pain intensity, bowel function recovery, and length of hospital stay. The components of the adapted ERAS protocols included opioid-free pain management, the avoidance of intra-abdominal drains, early feeding, and early mobilisation.

**Results:**

The implementation of modified ERAS protocols led to an improvement in clinical outcomes. The mean hospital stay was shorter in the ERAS group (12.07 compared with 16.47 days, *p* < 0.001). The timing of the first postoperative bowel movement differed significantly between the groups, with 93.3% of ERAS patients passing stool by postoperative day 2 compared with 15.6% in the non-ERAS group (*p* < 0.001). The mean Visual Analogue Scale score was lower in ERAS group on postoperative day 2 (3.83 compared with 5.47, *p* < 0.001). No increase in postoperative complications was observed in the ERAS group.

**Conclusion:**

The application of modified ERAS protocols was safe and effective for patients with ventral hernias after abdominal injuries and led to a reduced hospital stay, faster restoration of bowel function and decreased postoperative pain.

## Introduction

Due to ongoing military actions in Ukraine, there has been an increase in the number of wounded patients who have undergone surgery using the damage control surgery (DCS) approach following shrapnel wounds to the abdomen. DCS has become the gold standard for severe abdominal trauma [1, 2] and these operations are always performed as emergency procedures in accordance with the stages of medical evacuation and consist of three phases of treatment.

**FIGURE 1:**
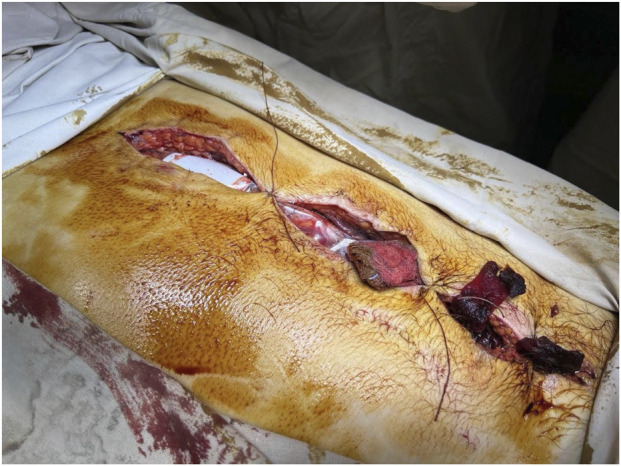
An example of temporary abdominal closure following the first stage of damage control surgery. *Photo taken by the authors. Licensed under CC-BY 4.0.*

In the first phase, surgery is performed with the goal of stopping bleeding and preventing contamination of the abdominal cavity, with temporary abdominal closure (Figure 1). This is followed by stabilization of the patient’s general condition and evacuation to the next level of medical care. Subsequently, the patient undergoes definitive surgery, which may include the formation of an anastomosis, creation of a stoma, and final haemostasis depending on the nature of the injury.

**FIGURE 2:**
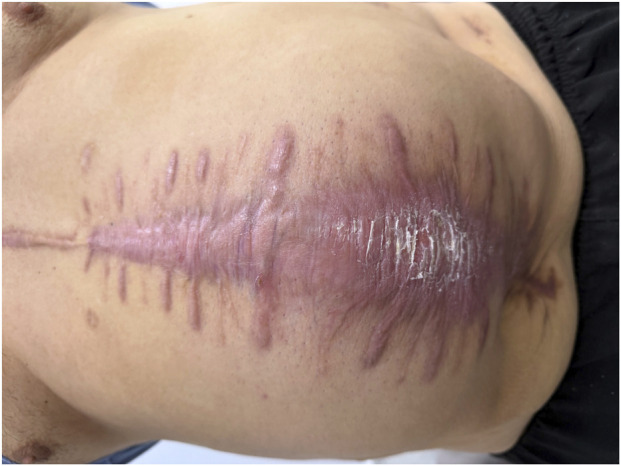
A patient with a large ventral hernia following an abdominal shrapnel wound. *Photo taken by the authors. Licensed under CC-BY 4.0.*

Such surgeries often result in serious anatomical disruptions of the anterior abdominal wall and complications such as secondary wound healing, and, as a consequence, lead to the formation of large ventral hernias in the future (Figure 2). The size of the hernia defect in all patients was classified according to the European Hernia Society (EHS) classification for incisional abdominal wall hernias, which defines defect width as W1 (<4 cm), W2 (≥4–10 cm), and W3 (≥10 cm) [3]. In this cohort, all patients presented with W3 hernia defects, which we refer to as large ventral hernias. Surgical repair of these patients is always challenging due to severe adhesions, tissue alterations, and changes in abdominal anatomy. The treatment of these patients is complex and aims to restore all functions as quickly as possible while minimising complications.

Enhanced Recovery After Surgery (ERAS) protocols have a strong evidence base for the management of colorectal and elective abdominal patients [4, 5]. However, their application in the treatment of patients with ventral hernias following DCS remains uninvestigated and requires proof regarding the safety of their use in such cases. This research aims to investigate the effectiveness and feasibility of applying adapted ERAS protocols to the treatment of patients operated on for ventral hernias following abdominal shrapnel wounds and damage control surgery.

## Materials and Methods

### Study Design and Setting

This retrospective cohort study was conducted at the Military Medical Clinical Centre of the Southern Region in Odesa, Ukraine, between June 2022 and April 2025. The study included 62 male patients who had sustained injuries and subsequently underwent elective surgery for ventral hernias. The minimum interval between the injury and the planned surgical procedure was 6 months. All the patients sustained small bowel injuries, with some of them having additional abdominal organ damage. The patients were compared in terms of body mass index (BMI) and age. Patients with significant comorbidities (diabetes mellitus, chronic cardiovascular insufficiency, chronic pulmonary disease, chronic kidney or liver disease, or chronic pain syndromes) were not included. Social habits (smoking, alcohol consumption) were recorded on admission. No active alcohol use was reported at the time of surgery. Smoking was common in the cohort but its prevalence was comparable between groups.

### Patient Groups

The patients were divided into two groups based on a chronological timeline. Both groups were comparable in terms of baseline clinical parameters, including BMI, comorbidities, previous surgical interventions, nature of injury, and hernia size (Table 1). Those who underwent surgery between June 2022 and September 2024 received standard care without the implementation of ERAS protocols. Patients operated on from September 2024 to April 2025 were managed according to adopted ERAS protocols. These are evidence-based, multimodal perioperative care pathways designed to reduce surgical stress, accelerate recovery, and improve clinical outcomes. They typically include preoperative patient education and optimization, minimization of fasting, standardized anaesthesia and analgesia, early mobilization, and early postoperative nutrition [6]. A total of 32 patients were treated in the non-ERAS group, while 30 patients received care under the ERAS protocol. In the ERAS group, compliance with the protocol was complete, with all key elements fully implemented in all patients.

**TABLE 1:** Demographic and baseline characteristics of the study population.

Parameter	ERAS group (n = 30)	Non-ERAS group (n = 32)
Age, years (median, range)	37.33 ± 7.51 years (range 25–52)	36.88 ± 7.44 years (range 23–51)
BMI, kg/m^2^ (median, range)	22.70 ± 2.68 (17.0–26.0)	22.73 ± 2.60 (18.5–27.0)
Smoking, n (%)	16 (53.3%)	17 (53.1%)
Primary trauma Type	Shrapnel wound to the abdomen	Shrapnel wound to the abdomen
Male sex, n (%)	30 (100%)	32 (100%)
Hernia defect size, cm, median (range)	12 (10.5–15)	11.8 (10.5–14)

### Preoperative Preparation, Surgical Approach and Recovery Protocol

In the preoperative period, the patients in the ERAS group, in contrast to those treated before the ERAS protocols introduction, did not undergo bowel preparation and were not subjected to preoperative fasting. Both groups received antibiotic prophylaxis 1 hour prior to the incision.

All hernia repairs were performed using an open approach with either an on-lay or a sub-lay (retromuscular) mesh placement. The choice of technique depended on the intraoperative condition of the abdominal wall. Although sub-lay repair is generally associated with favourable long-term outcomes in large ventral and incisional hernias [7], its use in this cohort was limited by the consequences of previous emergency operations. All patients had undergone multiple laparotomies after abdominal shrapnel injuries, which frequently resulted in severe adhesions and distortion of the posterior rectus sheath. In several cases, the retromuscular plane was partially or completely lost, making safe sub-lay dissection technically difficult or impossible. For this reason, sub-lay repair was performed only when the retromuscular anatomy was preserved. In patients with significant scarring or disruption of the posterior fascial layer, an on-lay technique was used as the safer and more feasible option. During surgery, a massive adhesive process was assessed using the Peritoneal Adhesion Index (PAI). The mean size of hernia defect that was measured as the greatest horizontal distance (width) between the lateral margins of the defect according to the EHS definition, was comparable between two groups.

The modified ERAS protocols included several key components. During surgery, intra-abdominal drains were generally avoided, urinary catheters were removed immediately after the operation, and nasogastric tubes were not used. Pain management in these patients was conducted without the use of opioid analgesics. Epidural catheters were placed for all patient groups in the operating room and were routinely maintained for three postoperative days according to the standard practice of our centre. In patients managed under the ERAS protocols, a minimal infusion of local anaesthetic through the epidural catheter allowed urinary catheters to be removed immediately postoperatively without complications. Also in the ERAS group, instead of postoperative opioids, patients received a transversus abdominis plane (TAP) block performed under ultrasound guidance in the operating theatre and repeated during the first three postoperative days as part of multimodal analgesia [8, 9], together with non-steroidal anti-inflammatory drugs (NSAIDs). In contrast, patients treated prior to the implementation of ERAS protocols received epidural analgesia followed by systemic opioid analgesics for up to 2 days postoperatively.

The patients in the ERAS group were allowed to drink water 1 hour after the surgery and to resume oral intake of food after 6 h. Early mobilisation was initiated on the day of surgery. Prior to the introduction of ERAS protocols, the patients were typically permitted to drink water only after 12 h, to begin eating after 24 h or later, and the mobilisation usually took place the day after surgery.

### Statistical Analysis

Statistical analysis was performed using IBM SPSS Statistics, version 27 (IBM Corp., Armonk, NY, United States). Continuous variables were assessed for normality using the Shapiro–Wilk test. As all continuous variables demonstrated normal distribution, they are presented as mean ± standard deviation and were compared using the independent samples t-test. Categorical variables were compared using the χ^2^ test or Fisher’s exact test when appropriate. All tests were two-sided, and *p*-values <0.05 were considered statistically significant. No missing data were present in the dataset.

### Data Collection

For the patients in both groups, the duration of surgery, the number and nature of postoperative complications, the recovery of bowel function (first bowel movement), the intensity of pain, and the length of hospital stay were recorded. An important feature of the length of hospital stay for military patients under current treatment conditions in Ukraine is that discharge is only possible after complete wound healing and suture removal, full recovery of all functions, and on the condition that the patient no longer requires any form of medical assistance. All the patients were informed about the nature of the operation and the postoperative period, and provided a written consent for the surgical procedure.

## Results

All the patients in both groups were male and had previously undergone surgery for abdominal shrapnel wounds. The two groups were comparable in age and BMI, with no statistically significant differences in baseline characteristics (Table 1). There were no patients with the significant comorbidities in either group. No active alcohol consumption was reported at the time of surgery. Smoking prevalence was 16 patients (53.3%) in the ERAS group and 17 patients (53.1%) in the non-ERAS group; the difference was not statistically significant (*p* = 1.00). The choice of surgical technique (on-lay or sub-lay), the severity of adhesions assessed by the PAI, hernia defect size, and duration of surgery were comparable between the groups. In patients treated under ERAS protocols, no drains were used, urinary catheters were removed immediately after the operation upon the patient’s return to the ward, and nasogastric tubes were avoided. In contrast, six patients in the non-ERAS group had intra-abdominal drains placed, and all patients in this group had urinary catheters and nasogastric tubes, which were removed only on the day following surgery (Table 2).

**TABLE 2:** Surgical characteristics and intraoperative details.

Parameter	ERAS group (n = 30)	Non-ERAS group (n = 32)
On-lay mesh repair, n (%)	13 (43.3%)	15 (46.9%)
Sub-lay mesh repair, n (%)	17 (56.7%)	17 (53.1%)
Duration of surgery, min	184.0 ± 27.4 min	183.0 ± 26.7 min
Severe adhesions (PAI >20), n (%)	7 (23.3%)	10 (31.3%)
Drain usage, n (%)	0 (0%)	6 (18.8%)
Urinary catheter use, n (%)	0 (0%)	32 (100%)
Nasogastric tube use, n (%)	0 (0%)	32 (100%)

### Duration of Hospital Stay

The mean duration of hospital stay in the ERAS group, taking into account the requirement that patients were discharged only after suture removal and full recovery, was 12.07 ± 2.30 days (ranging from 10 to 18 days). In the non-ERAS group, the average hospital stay was 16.47 ± 5.07 days (ranging from 10 to 24 days). This difference was statistically significant (*p* < 0.001).

### Seroma Formation

All the patients in the postoperative period underwent ultrasound examination of the anterior abdominal wall to detect the formation of seromas. Seroma formation was identified in 6 patients (20%) from the ERAS group and in 13 patients (40.6%) from the non-ERAS group (*p* > 0.05), indicating no statistically significant difference. These patients underwent ultrasound-guided seroma aspiration, and their hospital stay was extended until complete resolution of the seroma. When comparing patients who developed seromas with those who did not, there were no significant differences in surgical characteristics, including mesh position, size of the hernia defect, or the extent of subcutaneous dissection. Therefore, the lower seroma rate observed in the ERAS group is unlikely to be explained by differences in operative technique and may instead reflect factors associated with ERAS protocols, such as earlier mobilisation and faster postoperative recovery.

### Restoration of Bowel Function

The bowel function was assessed based on the return of peristalsis and the first postoperative bowel movement. In the ERAS group, 28 patients (93.3%) had their first bowel movement on postoperative day (POD) 2, while the remaining 2 patients (6.7%) passed stool on POD 3. In contrast, in the non-ERAS group, only 5 patients (15.6%) had their first bowel movement on POD 2; 19 patients (59.4%) passed stool on POD 3, and the remaining 8 patients (25.0%) on POD 4. The overall distribution of the timing of the first postoperative bowel movement differed significantly between the groups (*p* < 0.001).

In the non-ERAS group, one patient subsequently developed an early adhesive small bowel obstruction, which was successfully managed conservatively. This event occurred after the initial restoration of bowel function and therefore did not affect the recorded timing of the first postoperative bowel movement.

### Pain

The postoperative pain in the patients from both groups was assessed using the Visual Analogue Scale (VAS) on POD 1 and POD 2. In the group of patients treated prior to the implementation of ERAS protocols, the pain on the POD 1 was rated at 6.94 ± 1.05, and on the POD 2 at 5.47 ± 0.62, despite the use of epidural anaesthesia and opioids. In the ERAS group, where the patients received a TAP block and were prescribed NSAIDs, pain was rated at 6.17 ± 0.87 on the POD 1 and at 3.83 ± 0.75 on the POD 2. The differences in pain intensity between the groups were statistically significant on both POD 1 (*p* < 0.01) and POD 2 (*p* < 0.001). By the POD 3, both groups had reported similar pain levels, with an average score of 2.

### Non-Surgical Complications

No non-surgical complications occurred during hospitalization. Patients were followed for 6 months postoperatively, allowing assessment of early and intermediate outcomes, including hernia recurrence and reoperation; no recurrences or reoperations were reported during this period.

A comparative summary of all postoperative outcomes is provided in Table 3.

**TABLE 3:** Postoperative outcomes comparison.

Parameter	ERAS group (n = 30)	Non-ERAS group (n = 32)	p-value
Hospital stay, days (mean ± SD, range)	12.07 ± 2.30 (10–18)	16.47 ± 5.07 (10–24)	<0.001
First postoperative bowel movement, n (%) • POD 2 • POD 3 • POD 4	28 (93.3%) 2 (6.7%) 0	5 (15.6%) 19 (59.4%) 8 (25%)	<0.001
VAS pain score, mean ± SD • POD 1 • POD 2	6.17 ± 0.87 3.83 ± 0.75	6.94 ± 1.05 6.94 ± 1.05	<0.01 <0.001
Postoperative morbidity, n (%) • Seroma formation • Small bowel obstruction	6 (20%) 6 (20%) 0	14 (43.8%) 13 (40.6%) 1 (3.1%)	>0.05 >0.05 = 1.00

## Discussion

This study evaluated the introduction of an ERAS pathway for elective ventral hernia repair in patients with a history of abdominal shrapnel wounds who had undergone multiple emergency laparotomies managed according to DCS principles [1, 2]. Evidence for ERAS in abdominal wall reconstruction is limited, as most studies focus on elective, non-trauma populations [4]. Our findings therefore add data to this under-researched area.

A clear reduction in the length of hospital stay was seen in the ERAS group. This finding is consistent with reports from other abdominal procedures, where structured perioperative pathways are repeatedly shown to support faster recovery [4–6]. Early oral feeding and early mobilisation, key elements of ERAS, are known to promote the restoration of normal gastrointestinal function, which corresponds with the earlier return of bowel activity seen in our cohort.

Postoperative pain scores were also improved in the ERAS group. The combination of TAP block and NSAIDs provided effective analgesia without the need for opioids, even in patients with a history of multiple operations. This is compatible with existing evidence showing that TAP block reduces postoperative pain and opioid requirements after abdominal wall surgery [8, 9].

Both on-lay and sub-lay repairs were used according to intraoperative findings, and their distribution was comparable between groups. The abdominal wall literature outlines the advantages of retromuscular mesh placement but also notes its limitations in cases with altered anatomy or dense adhesions, which are common after trauma and repeated laparotomies [3, 7]. The seroma rate in this study fell within the expected range after open ventral hernia repair. Although slightly lower in the ERAS cohort, no significant difference was observed. Current evidence does not demonstrate a direct relationship between ERAS implementation and seroma formation; however, earlier mobilisation and faster restoration of normal physiology may facilitate postoperative fluid resorption [4, 6]. Further prospective studies are required to clarify this association.

This population has several important characteristics. All patients had sustained abdominal shrapnel injuries and had undergone urgent procedures in accordance with DCS principles [1, 2]. The resulting adhesions, scarring, and trauma-related distortion of the abdominal wall make subsequent elective reconstruction technically challenging. The present findings demonstrate that an ERAS pathway can be applied safely even in this demanding context and may offer meaningful benefit despite the complexity of the cases.

### Strengths

This study addresses a patient group rarely represented in ERAS research and provides insight into the management of ventral hernias following trauma and multiple laparotomies. Baseline comparability between groups strengthens the validity of the findings. Full adherence to the ERAS pathway enabled assessment of the complete protocol. Objective outcomes, including pain scores, return of bowel function, and postoperative complications, enhance the reliability of the results.

In addition, the study contributes evidence in a population that has not been comprehensively described in the ERAS literature, supporting the relevance and novelty of the findings.

### Limitations

Several limitations must be considered. The cohort was relatively small, which reduces the ability to detect differences in less common postoperative outcomes. Although full adherence to the ERAS pathway was achieved in the ERAS group, the introduction of the protocol may have led to broader changes in perioperative practice beyond the specific ERAS elements, which could affect the comparability of the two periods. Relevant covariates such as smoking status and chronic pain were collected, and no active alcohol use was reported at the time of surgery. However, these factors were not incorporated into an adjusted statistical analysis, which limits the ability to assess their potential influence on outcomes.

The length of hospital stay in this military setting differs from standard practice in other healthcare systems, which restricts external generalisability. As a single-centre retrospective study, the design also carries a risk of temporal changes in perioperative practice influencing outcomes. Long-term follow-up, including recurrence rates, was not available.

### External Validity and Future Directions

Despite these limitations, many elements of the ERAS pathway applied here are transferable to other centres managing elective ventral hernia repair. Future work should include prospective studies with larger samples, longer follow-up, and incorporation of patient-reported outcomes. Further development and optimisation of ERAS components may enhance postoperative recovery and strengthen the role of ERAS in abdominal wall reconstruction.

## Conclusion

The application of ERAS protocols has proven effective across various surgical disciplines. In this study, the use of modified ERAS protocols demonstrated a positive impact on the patients with large ventral hernias following previous abdominal trauma managed with DCS. The implementation of these protocols was associated with a reduced hospital stay, faster patient recovery, and minimised postoperative pain.

No adverse effects on postoperative outcomes were observed as a result of introducing the modified ERAS protocols in this patient cohort.

## Data Availability

The original contributions presented in the study are included in the article/supplementary material, further inquiries can be directed to the corresponding author.
